# MY MESSAGE: CUSTOMIZED VISUAL CUES FOR PERSONS WITH DEMENTIA USING ADAPTIVE DISPLAY TECHNOLOGY

**DOI:** 10.1093/geroni/igz038.001

**Published:** 2019-11-08

**Authors:** Michael Skrajner, John Zeisel, gregg Gorzelle, Tom Albright, Sergei Gepshtein

**Affiliations:** 1 Hearthstone Alzheimer Care, Woburn, Massachusetts, United States; 2 Hearthstone, Woburn, Massachusetts, United States; 3 Salk Institute, La Jolla, California, United States

## Abstract

The My MESSAGE™ (MM) intervention visually conveys individualized messages to persons with dementia (PWD) on adaptive electronic displays in strategic locations activated by an approaching individual. MM aims to improve quality of life (QoL) and reduce challenging behaviors, especially repetitive questioning and wayfinding difficulties. For example, for a PWD who repeatedly asks when his/her daughter will visit, the monitors display a reassuring message, such as, “I love you and will visit soon.” A PWD who has trouble finding his/her bedroom, receives directions on the monitor. MM was tested in a Phase 1 STTR. The sample consisted of 22 PWD: 10 in Experimental Group (EG) and 12 in Control Group (CG). MM had positive immediate impacts on EG participants, with 100% exhibiting positive affect, 80% reduced agitation, 60% reduced anxiety, and 50% improved mood. MM had positive longer-term/generalized impacts on PWD, as well. For the CG, QoL (based on the Dementia Quality of Life Scale) tended to decline from baseline to post-treatment for the CG (p=0.053); with no decline in the EG. Agitation (based on the Cohen-Mansfield Agitation Inventory) tended to increase in the CG (p=0.057); no change was found in the EG. There was a significant decrease in neuropsychiatric symptoms (based on the Neuropsychiatric Inventory-Nursing Home) for the EG (p=.040); no change was detected for the CG. The results suggest that MM is worthy of further development and evaluation in a planned Phase 2 study.

